# Towards Mapping Mouse Metabolic Tissue Atlas by Mid‐Infrared Imaging with Heavy Water Labeling

**DOI:** 10.1002/advs.202105437

**Published:** 2022-03-23

**Authors:** Xinwen Liu, Lixue Shi, Lingyan Shi, Mian Wei, Zhilun Zhao, Wei Min

**Affiliations:** ^1^ Department of Chemistry Columbia University New York NY 10027 USA

**Keywords:** heavy water labeling, infrared imaging, metabolic heterogeneity, metabolism, multivariate analysis

## Abstract

Understanding metabolism is of great significance to decipher various physiological and pathogenic processes. While great progress has been made to profile gene expression, how to capture organ‐, tissue‐, and cell‐type‐specific metabolic profile (i.e., metabolic tissue atlas) in complex mammalian systems is lagging behind, largely owing to the lack of metabolic imaging tools with high resolution and high throughput. Here, the authors applied mid‐infrared imaging coupled with heavy water (D_2_O) metabolic labeling to a scope of mouse organs and tissues. The premise is that, as D_2_O participates in the biosynthesis of various macromolecules, the resulting broad C‐D vibrational spectrum should interrogate a wide range of metabolic pathways. Applying multivariate analysis to the C‐D spectrum, the authors successfully identified both inter‐organ and intra‐tissue metabolic signatures of mice. A large‐scale metabolic atlas map between different organs from the same mice is thus generated. Moreover, leveraging the power of unsupervised clustering methods, spatially‐resolved metabolic signatures of brain tissues are discovered, revealing tissue and cell‐type specific metabolic profile in situ. As a demonstration of this technique, the authors captured metabolic changes during brain development and characterized intratumoral metabolic heterogeneity of glioblastoma. Altogether, the integrated platform paves a way to map the metabolic tissue atlas for complex mammalian systems.

## Graphical abstract

An integrated platform combining FTIR imaging, heavy water labeling and advanced multivariate analysis is developed to pave a way to map the metabolic tissue atlas of complex mammalian systems. Specifically, metabolic signatures at both inter‐organ and intra‐tissue levels are discovered. Moreover, metabolic profile changes during brain development are captured and intratumoral metabolic heterogeneity of glioblastoma is revealed in situ.

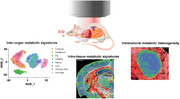

## 1 Introduction

Metabolism and genetics are two defining features of life. After all, metabolism is regulated by gene expressions; in turn, gene expressions require continuous supplies of proper metabolites.^[^ ^1^ ^]^ These two processes coordinate together to achieve homeostasis. Metabolism is also an important theme in medicine and pharmacology. A series of diseases, including diabetes,^[^ ^2^ ^]^ atherosclerosis,^[^ ^3^ ^]^ and cancers,^[^ ^4^ ^]^ have close connections to metabolic aberrations. Clarifying metabolic pathways also provides crucial targets for drug therapy.^[^ ^5^ ^]^

In the context of complex mammals, metabolism arises within individual cells depending on the cell type, is balanced through cell–cell interaction and metabolite sensing in tissues, and is further regulated through inter‐organ communications.^[^ ^6^ ^]^ Therefore, metabolic activities are expected to be closely associated with organs, tissues, and cell states, and should further respond to the developmental stages and disease progresses. Thus, metabolism‐targeted tissue atlas will be a valuable resource to understand tissue biology from a system level. Such metabolic tissue atlas will also be complementary to the prevailing genetics‐targeted tissue atlas.

Despite its perceived importance, how to capture organ‐, tissue‐, and cell‐type‐specific metabolic profiles (i.e., metabolic atlas) in situ is a challenging task, largely due to the lack of metabolic imaging tools with high resolution and high throughput. Positron emission tomography and magnetic resonance spectroscopy lack sufficient spatial resolution to provide cellular spatial information.^[^ ^7^ ^]^ Fluorescence microscopy requires labeling with bulky fluorophores, which can easily perturb normal functions of small metabolites in vivo. Photoacoustic imaging can achieve high penetration depth, but its spatial resolution is less ideal and the choice of metabolites is limited by either the confined endogenous chromophores or the bulky exogenous contrast agents.^[^ ^8^ ^]^ Mass spectrometry imaging is destructive and time‐consuming, especially for mapping mammalian tissues.^[^ ^9^ ^]^ Raman imaging coupled with Raman‐active probes is a powerful platform of metabolic imaging.^[^ ^10^ ^]^ Nonetheless, Raman‐based metabolic imaging is less ideal for spatial‐spectral scanning of large tissue specimens, due to the rather slow imaging speed (e.g., spontaneous Raman) or limited spectral coverage (e.g., stimulated Raman). Overall, a high‐throughput (implying both fast imaging speed and broad spectral coverage) metabolic imaging platform with cellular spatial resolution is in demand for building metabolic atlas.

Recently, a new framework of mid‐infrared (MIR) metabolic imaging was developed by coupling MIR microscopy with infrared (IR)‐active vibrational probes, which allows large‐area metabolic imaging with cellular‐level spatial resolution, rich spectral information, and high imaging throughput.^[^ ^11^ ^]^ In particular, by using heavy water (D_2_O) as a universal metabolic probe, protein synthesis and lipid synthesis of tissue specimens were imaged with Fourier transform infrared (FTIR) microspectroscopy. While promising, the prior method only studied two signature C─‐D peaks and did not use the full spectral information of D_2_O‐derived broad C—D peaks spanning around 200 wavenumbers in the IR spectrum, thus not compatible with the concept of metabolic profiling which is of multiparameter inherently. Moreover, with the limited parameters and thus less‐optimal sensitivity of this prior method, it remains unclear whether it can capture the organ‐, tissue‐ and cell‐type‐specific metabolic signatures.

Here, we present a next‐generation platform that integrates FTIR imaging, IR‐active vibrational probes, and advanced multivariate analysis to systematically map metabolic signatures at both inter‐organ and intra‐tissue levels. Our key premise is that, as D_2_O participates in the biosynthesis of various macromolecules (including lipids, proteins, DNA/RNA, and carbohydrates), the resulting broad C—D spectrum should interrogate a wide range of metabolic pathways, making it compatible with the concept of metabolic profiling and hence is very likely to reflect organ‐, tissue‐ and cell‐type‐specific metabolic signatures. To extract the hidden information in the broad C—D spectrum, we employed advanced multivariate analysis including hierarchical cluster analysis (HCA), t‐distributed stochastic neighbor embedding (t‐SNE)^[^ ^12^ ^]^ and uniform manifold approximation and projection (UMAP)^[^ ^13^ ^]^ to analyze the entire C—D spectrum instead of only focusing on two C—D peaks. A large‐scale metabolic atlas map of various organs was generated for the first time, revealing distinctive metabolic profiles of each organ. Moreover, leveraging the power of clustering methods, we discovered metabolic signatures with cellular spatial resolution from tissues, which exhibited high correlations to their anatomical features. To demonstrate the utility of our platform, we compared the clustering results from pup and adult mice and captured the metabolic profile changes during brain development. Furthermore, intratumoral metabolic heterogeneity of glioblastoma was also revealed, with the periphery of the tumor being metabolic distinct from the core of glioblastoma. Overall, the integrated platform has great potential to map the metabolic atlas of mammalian systems ranging from cellular scale, tissue‐scale, to organ‐scale.

## 2 Results

### 2.1 D_2_O as a Metabolic Probe for FTIR Tissue Imaging

**Figure 1:**
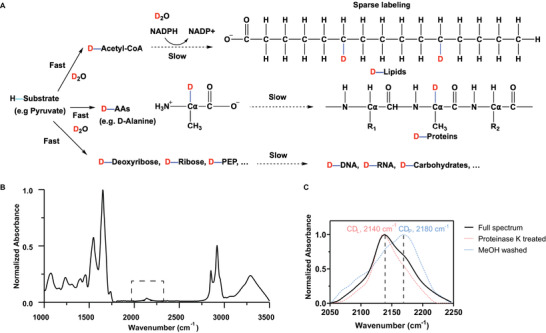
Heavy water as a metabolic probe for FTIR tissue imaging. A) Heavy water labeling mechanism. Due to the sparse labeling pattern of heavy water, the position of deuterium labeling on lipids is random. This sketch only represents one possible labeling pattern. B) A typical normalized IR absorption spectrum of cerebellum tissue samples from mice fed with 25% D_2_O in drinking water. The associated broad peak of D_2_O labeling is highlighted with a dashed rectangle in the cell‐silent region. C) IR absorption CD spectra of cerebellum tissue treated with proteinase K digestion and methanol wash, revealing CD_L_ and CD_P_ components.

As the ubiquitous solvent of life, water diffuses freely across cell and organelle membranes and participates in the synthesis of virtually all the small and large biomolecules.^[^ ^14^ ^]^ Therefore, D_2_O, which is a well‐known isotopologue of water, could be a universal tracer for monitoring the de novo synthesis of a diverse range of biomolecules.^[^ ^15^ ^]^ In principle, D_2_O labels biomolecules through enzymatic incorporation under physiological conditions^[^ ^16^ ^]^ (**Figure** **1**A). By replacing the original C—H bonds with C—D bonds, deuterium quickly labels metabolic precursors including acetyl‐CoA, non‐essential amino acids, deoxyribose, and phosphoenolpyruvate. These precursors are then slowly converted into macromolecules such as lipids,^[^ ^17^ ^]^ proteins,^[^ ^18^ ^]^ DNA/RNA,^[^ ^19^ ^]^ and carbohydrates,^[^ ^20^ ^]^ relying on cellular metabolic reactions.

D_2_O is a desired probe for tissue metabolic profiling with FTIR microscopy for several reasons. By tracing the emergence of the broad C—D peak (referred to as the C—D region, 2050–2250 cm^−1^) from the original O—D bond, D_2_O labeling can not only reflect the general metabolic rate but also reveal macromolecule‐specific metabolic dynamics. Of note, the C—D region lies in the cell‐silent window (1800–2700 cm^−1^) of the IR spectrum (Figure 1B), which avoids the perturbance of vibrational transitions of endogenous biomolecules. Moreover, D_2_O is uniquely suitable for animal labeling, which is simple, safe, and economic. In fact, D_2_O delivery and labeling for mice could be performed directly through D_2_O administration in their drinking water.

Experimentally, a typical IR spectrum of cerebellum tissue collected from mice fed with 25% D_2_O is shown in Figure 1B. This D_2_O concentration was chosen as a good balance between the in vivo C—D signal strength and the concern of mice safety. Since 20% D_2_O enrichment in mice body water has not caused any side effects for physiological processes,^[^ ^21^ ^]^ the chosen administration concentration of 25% D_2_O in drinking water to mice corresponds to a 15–17.5% enrichment in body water, which is lower than the 20% safe limit.^[^ ^14^ ^]^ Compared with the label‐free control spectrum (Figure S4, Supporting Information), the reflected C—D signal in the IR spectrum appears as a broad peak at around 2140 cm^−1^ in the highlighted C—D region, together with a shoulder peak at 2180 cm^−1^. As demonstrated in the previous study,^[^ ^11^ ^]^ after treating the tissue slices with either proteinase K to digest protein or methanol to dissolve lipids, these two signature peaks were confirmed to be attributed to C—D containing lipids and proteins respectively. Coupled with linear unmixing algorithms, we further extracted D_2_O‐derived protein synthesis signal (CD_P_, 2180 cm^−1^) and de novo lipid synthesis signal (CD_L_, 2140 cm^−1^) from IR spectra (Figure 1C). As a result, protein and lipid synthesis activities can be measured in situ through the ratio plots of CD_P_/amide and CD_L_/CH_2_, respectively. Hereinafter we refer to this type of characterization method, which mainly focused on the two frequencies of CD_L_ and CD_P_ in the C—D spectrum, as the bivariate analysis method.

### 2.2 Multivariate Analysis Reveals Unique Metabolic Profiles of Each Organ

Characterizing the metabolic difference and relations of multiple organs is of great importance, as each organ is expected to have its own metabolic function while various organs coordinate together to achieve whole‐body energy homeostasis. However, the bivariate analysis alone, which only focuses on two frequencies of CD_L_ and CD_P_, is not compatible with the concept of metabolic profiling. After all, the signal associated with D_2_O labeling in the IR spectrum is a broad peak consisting of various wavenumbers rather than two components. Moreover, considering that D_2_O is a universal tracer, it is very likely that more macromolecule‐specific pathways are reflected in the hyperspectral data of the C—D region. We hypothesize that multivariate analysis that covers all the wavenumbers in the entire C—D region (2050–2250 cm^−1^) can provide more abundant statistical information as well as higher sensitivity and broader coverage of metabolic pathways. Thus, multivariate analysis has great potential to distinguish the metabolic status of different organs.

**Figure 2:**
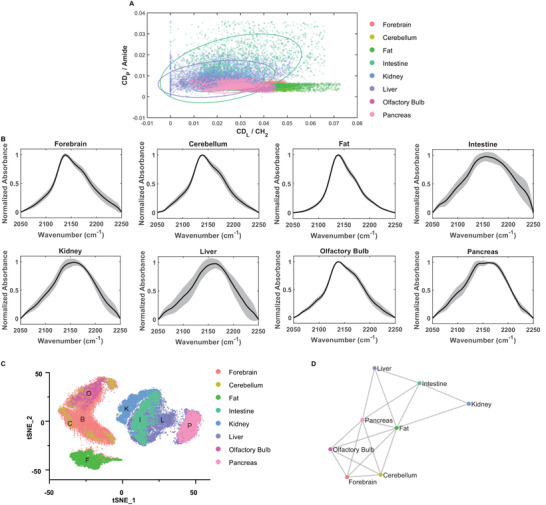
Multivariate analysis reveals the organ‐specific metabolic profiles. A) Bivariate characterization: 2D scatter graph of CD_L_/CH_2_ and CD_P_/amide for FTIR images of eight organs from the same D_2_O fed mouse (P35), which failed to distinguish the metabolic difference among most of the organs. Solid lines are error ellipses with 99% confidence. Each organ has 5000 randomly selected ratio values. B) Normalized average C—D spectra of mouse organs. The shaded area indicates the standard deviation of all pixels in the images. C) t‐SNE map of the mouse organ C—D spectra. 5000 spectra of each organ are selected as the input data. B represents the forebrain; C represents the cerebellum; F represents fat; I represents the intestine; K represents the kidney; L represents the liver; O represents the olfactory bulb; P represents the pancreas. D) Pairwise Spearman correlation network of the C—D spectra of mouse organs.

We collected the FITR images of eight different organs and tissues (Figure S1, Supporting Information) from the same D_2_O fed mice, including the forebrain (without the olfactory bulb), cerebellum, adipose (fat), intestine, kidney, liver, olfactory bulb, and pancreas. To compare the performances between bivariate analysis and multivariate analysis method, we first applied the bivariate analysis to plot the 2D scatter graph of CD_L_/CH_2_ and CD_P_/amide over the pixels in each organ's FTIR image. These two ratio values of 5000 pixels were randomly selected from each organ's image for demonstration. Altogether, eight organ‐related distributions were presented simultaneously in **Figure** **2**A. It can be observed that the distributions of different organs were highly overlapped, suggesting that bivariate analysis alone is not informatic enough for metabolic phenotyping. We also performed analysis of variance (ANOVA) tests for the groups of either CD_L_/CH_2_ or CD_P_/amide ratio value for eight tissues, whose P values were listed in Table S1, Supporting Information. For CD_L_/CH_2_, adipose, kidney, and pancreas are not significantly different; for CD_P_/amide, the brain organ (including the forebrain, cerebellum, and olfactory bulb) is indistinguishable from adipose and pancreas. Intestine and liver cannot be told apart for both groups. These results further confirmed the insensitivity of analytical methods towards metabolic phenotyping when only focusing on one or two signature C—D peaks.

We then turned to multivariate analysis. The average spectrum in the entire C—D region of each tissue was extracted with its standard deviation (Figure 2B), demonstrating varied C—D profiles in tissues from different organs. Considering the universality of D_2_O labeling, we refer to the entire C—D profile as the metabolic profile here, which potentially reflects the metabolic status for tissues and organs. To further characterize the metabolic profiles of multiple organs, t‐SNE, a nonlinear dimension reduction method with popularity in the gene‐sequencing community, was initiatively applied to the hyperspectral data in the C—D region. Specifically, 5000 spectra were randomly selected from the pixels in the FTIR image of each tissue slice. Overall, 40 000 (i.e., 5000 × 8) C—D metabolic profiles were used as the input of t‐SNE. Interestingly, six clusters representing six different organs appeared in the t‐SNE plot (Figure 2C), which proves the unique metabolic profiles of each organ. Specifically, the forebrain, cerebellum, and olfactory bulb crowd together to form a large cluster, which agrees well with their anatomical origins. Besides the brain cluster, the other five organs are well‐separated, which demonstrates the higher sensitivity of multivariate analysis towards metabolic phenotyping compared with the bivariate analysis. More details can also be visualized from the t‐SNE plot. For instance, the forebrain has a broader distribution in terms of their metabolic profiles while the cerebellum and olfactory bulb have relatively centered distributions. Moreover, it is observed that the kidney, intestine, liver, and pancreas are more closely distributed, especially for the intestine and the liver. This result is consistent with the connected metabolic functions of these two organs, especially for lipid metabolism.^[^ ^22^ ^]^

We also calculated the Spearman correlation coefficients of the metabolic profiles from different organs and generated a correlation network (Figure 2D). The network topology further elucidates the correlations of metabolic profiles of different organs, such as the strong correlation of forebrain, cerebellum, and olfactory bulb, the close connection between liver and intestine, as well as the intermediate roles of adipose and pancreas connecting two ends in the network. Hence, t‐SNE based multivariate analysis not only reveals the distinct metabolic profiles of each organ but also demonstrates the distributions and correlations of these metabolic profiles, which are related to their anatomical origins and metabolic functions. Considering that metabolism is regulated through the orchestration of multiple organs,^[^ ^22^ ^]^ our results might reflect the underlying metabolic connections of these organs to some extent.

### 2.3 Spatially Mapping Intra‐Tissue Metabolic Signatures through HCA Clustering

Multivariate analysis, which utilizes the entire C—D spectra, has revealed the distributions and correlations of metabolic profiles between different organs. However, tissues such as the brain have complicated histology and contain various cell types. It is of great importance to further identify metabolic signatures of refined histological structures within tissues. Therefore, a question to ask is whether intra‐tissue metabolic signatures can also be revealed. To that aim, we turned to HCA clustering, which is broadly adopted in label‐free IR imaging to identify histological patterns inside tissues.^[^ ^23^ ^]^ Compared to other common unsupervised learning methods such as k‐means, HCA provides a dendrogram that represents the dissimilarity between clusters, which gives useful guidance to the number of clusters to be assigned. Thus, HCA was selected as our clustering method.

**Figure 3:**
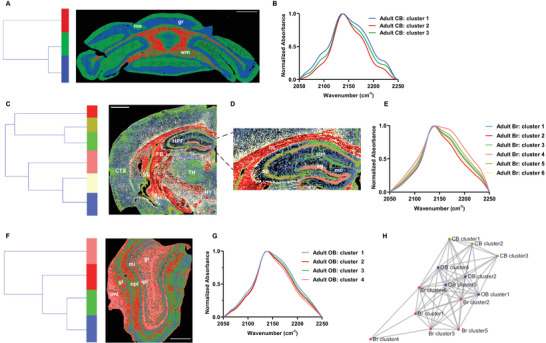
Mapping intra‐tissue metabolic signatures of mouse tissues through HCA clustering. A) HCA clustering of a cerebellum tissue section from adult mice, where mo represents the molecular layer; gr represents the granule layer; wm represents the white matter. Scale bar: 1mm B) Spectral centroids from Figure 3A. CB represents the cerebellum. C) HCA clustering of a forebrain tissue section from adult mice. CTX indicates cerebral cortex; FB indicates fiber tracts; HPF indicates hippocampal formation; HY indicates hypothalamus; TH indicates thalamus; VL indicates lateral ventricle. Scale bar: 1 mm. D) Amplified forebrain hippocampus region from Figure 3C, where cc denotes the corpus callosum; mo denotes the molecular layer of dentate gyrus; sg denotes the granule layer of dentate gyrus; slm denotes the stratum lacunosum‐moleculare; sp denotes the pyramidal layer. E) Spectral centroids from Figure 3C. Br represents the forebrain. F) HCA clustering of an olfactory bulb tissue section from adult mice, where gl represents the glomerular layer; gr represents the granule layer; ipl represents inner plexiform layer; mi represents the mitral layer; onl represents the olfactory nerve layer; opl represents the outer plexiform layer. Scale bar: 500 µm. G) Spectral centroids from Figure 3F. OB represents the olfactory bulb. (H) Pairwise Spearman correlation network of segmented clusters in Figure 3A,C,F.

By applying HCA to the C—D region of tissue sections from adult mice, we successfully revealed metabolic activity‐defined patterns in situ, together with their distinct metabolic profiles, in the cerebellum, brain, and olfactory bulb (**Figure** **3**). The clustering patterns show impressively high correlations to their anatomical features. For instance, we clearly classified layered metabolic patterns in the cerebellum (Figure 3A), which agrees well with its three anatomical layers including molecular layer, granule layer, and white matter. In fact, the three partitioned clusters from the HCA dendrogram exactly represent these three anatomical layers (Figure 3A), suggesting distinct metabolic activities for each anatomical layer. The metabolic signatures of anatomical layers are further reflected in their corresponding cluster centroids (Figure 3B). For instance, the granule layer has a higher shoulder peak at around 2180 cm^−1^ (contributed mainly from newly synthesized proteins, Figure 1C) in its C—D spectrum compared with the other two layers, which indicates the relatively active protein synthesis in this region and is consistent with the reported higher protein synthesis of the granule layer compared with the other regions inside rat cerebellum (P35).^[^ ^24^ ^]^ Hence, each histological structure has its unique metabolic profile and has been successfully distinguished by HCA. We also analyzed the forebrain and olfactory bulb using the same strategy. Remarkably, more detailed structures were revealed with specific metabolic information. For instance, in the forebrain, fiber tracts were well separated from the lateral ventricle (Figure 3C,E). Cerebral cortex layering was also mapped out with different metabolic profiles. Furthermore, the refined anatomical features within hippocampus formation (HPF) were unveiled (Figure 3D). The granule cell layer of the dentate gyrus (DG‐sg) demonstrated a unique high shoulder peak related to the newly synthesized proteins compared with all the other clusters, indicating its relatively vigorous protein synthesis. The pyramidal layer (sp) of CA1 and CA3 were separated, where CA1sp shares the same cluster assignment with the DG‐sg while CA3sp belongs to another cluster showing a different metabolic profile. The same cluster assignment of the CA1sp and the DG‐sg is consistent with the reported protein metabolism in the hippocampus, where the most active protein synthesis signal was found in both the granule cells in the dentate gyrus and the pyramidal cells in the CA1 region,^[^ ^25^ ^]^ suggesting the effectiveness of our analysis method. For the olfactory bulb, histological landmarks were also well‐separated (Figure 3F). Interestingly, the granule layer (gr) and olfactory nerve layer (onl) have the same cluster assignment, which might indicate the similar metabolic activities between these two refined structures.

As the cerebellum, forebrain, and olfactory bulb all originate from the same nervous system, we further constructed a network characterizing the correlation of the C—D spectra of each segmented cluster among these tissue samples (Figure 3H). Clusters from the olfactory bulb seem to function as intermediaries connecting the clusters in the forebrain and cerebellum, which might suggest their metabolic similarity or connections. Moreover, forebrain cluster 4, which locates at the DG‐sg and the CA1sp, asides far away from all the other clusters. This indicates that cells in these anatomical structures have a unique metabolic profile with reflected active protein synthesis even in the entire analyzed brain regions.

A technical point worth discussing here is that we normalized the entire C—D spectra and used it as the input for HCA clustering. Interestingly, despite the presence of intensity information in the data before C—D normalization, the HCA clustering result of unnormalized C—D data is not as good as that of normalized C—D data. For instance, when applying HCA on unnormalized C—D data of the adult cerebellum, it failed to identify the molecular layer from white matter (Figure S2, Supporting Information). A possible explanation is that the intensity information dominates the unnormalized data, which can overwhelm subtle differences of metabolic profiles. Normalization of the C—D region helps to uncover the different metabolic profiles of various histological features.

### 2.4 Capturing the Metabolic Profile Changes during Brain Development

**Figure 4:**
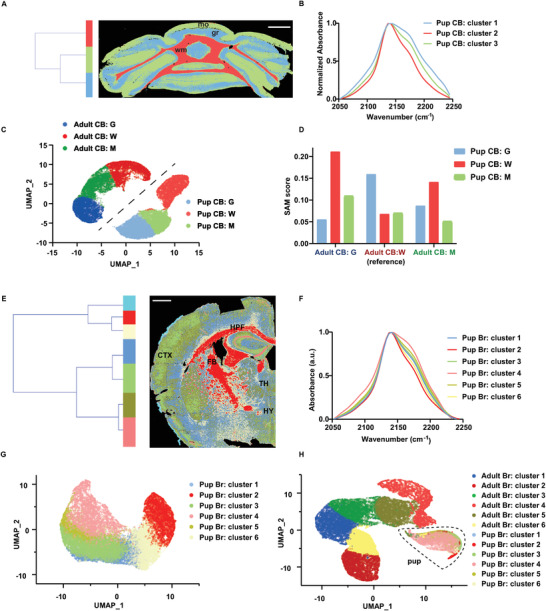
Capturing metabolic profile changes during mouse brain development. A) HCA clustering of the cerebellum tissue section from pup mice. Scale bar: 1mm. B) Spectral centroids from Figure 4A. CB represents the cerebellum. C) UMAP overlay of segmented clusters of cerebellum tissue sections from both adult and pup mice. 5000 spectra of each cluster (from Figures 3A and 4A) are used as the input. G represents the granule layer; W represents white matter; M represents the molecular layer. D) SAM score boxplot of the C—D spectra of cerebellum landmarks from both pup and adult mice. The C—D spectra of adult mice are used as the reference spectra here. E) HCA clustering of the forebrain tissue section from pup mice. Scale bar: 1 mm. F) Spectral centroids from Figure 4E. Br represents the forebrain. G) UMAP overlay of segmented clusters of forebrain tissue sections from pup mice only (Figure 4E). 5000 spectra of each cluster are used as the input. H) UMAP overlay of segmented clusters of forebrain tissue sections from both adult and pup mice. 5000 spectra of each cluster (from Figures 3C and 4E) are used as the input.

With the establishment of this new platform, we now pursue the demonstration of its utility. The very high demand for energy and fast rates of ATP formation and utilization during brain development is tightly associated with various metabolic activities.^[^ ^26^ ^]^ To further explore this process, we collected FTIR imaging data of brain tissue sections from pup mice besides adult mice. Applying the same analysis workflow, anatomical structures of the cerebellum and the forebrain from pup mice were revealed with specific metabolic profiles (**Figure** **4**). For the latter dataset, we noticed that cluster 7 (cyan color label) from HCA clustering only highlighted the very edges of the pup forebrain with abnormal C‐D spectra, which is likely caused by some artifact introduced during the tissue air‐drying process. Thus, data from cluster 7 were excluded in the following analysis. Overall, both the pup and adult cerebellum were classified into three clusters matching the three anatomical layers. Meanwhile, both the pup and adult forebrain were classified into six biologically relevant clusters with very similar spatial patterns, which demonstrates the consistency of our analysis approach.

To further explore the metabolic connection between pup and adult datasets, cerebellums were used as the benchmark samples, where 5000 C—D spectra were randomly selected from each segmented cluster of both the pup mice (Figure 4A) and the adult mice (Figure 3A), making up an integrated dataset of 30 000 (5000 × 3 × 2) spectra. Interestingly, when applying UMAP on this integrated dataset, which is computationally more efficient and can better preserve the global structure compared with t‐SNE,^[^ ^27^ ^]^ six clusters were formed representing the three anatomical layers of the cerebellum from both pup and adult mice (Figure 4C). To better differentiate these two datasets, color coding with dark shade was used to represent clusters from adult mice and colors with light shade indicated clusters from pup mice. The result is quite informative. Not only can it demonstrate the distribution of these three anatomical layers within the same tissue specimen, but also it shows the metabolic connection of these layers between the pup and the adult mice. Nearly one‐to‐one correspondence between these layers was spatially mapped, such as the apparent closer distribution between the granule layer in the adult mice (dark blue) and the granule layer in the pup mice (light blue), which might indicate their metabolic similarity. To further test the similarity of metabolic profiles along the same anatomical layer of the cerebellum during brain development, we calculated the spectrum angle mapper (SAM) scores, which has been widely used as a spectrum similarity measure,^[^ ^28^ ^]^ of the cluster centroids (Figure 4D). Specifically, the cluster centroids from the adult mice were used as the reference spectra while those from the pup mice were used as the test spectra. Using the granule layer as an example, when the metabolic profile from the adult granule layer is used as the reference, the metabolic profile of the pup granule layer showed the lowest SAM score compared with the other two pup anatomical layers. As a lower SAM score represents higher spectral similarity, these results directly proved the high similarity of metabolic profile among the same cerebellar anatomical layers during brain development.

We also applied the same analytical strategy to forebrain tissue sections from both pup and adult mice (Figure 4E–H). Interestingly, when exclusively analyzing the pup mice dataset with UMAP, these six clusters were successfully separated (Figure 4G). However, when analyzing both pup and adult mice datasets together, all clusters from the pup forebrain converged into a single region in the UMAP plot (Figure 4H). These two results together suggest that the metabolic profiles of histological markers in the adult brain are more distinct from each other while those in the pup brain are more similar to each other. In other words, the metabolic heterogeneity over different regions in the adult brain is more evident in comparison with the pup brain, suggesting the metabolic heterogeneity of brain structures has been increased over time during this development process. This is different from what was observed in the cerebellum, where anatomical layers in both pups and adults were clearly separated (Figure 4C). Together, these results might suggest that the cerebellum reaches the matured metabolic status in an earlier stage compared with the forebrain, which is consistent with the facts that the mouse hindbrain usually develops faster than the forebrain^[^ ^29^ ^]^ and that the cerebellum reaches maturation faster against other regions in the forebrain (e.g., dentate gyrus).^[^ ^30^ ^]^

### 2.5 Revealing Intratumoral Metabolic Heterogeneity of Glioblastoma In Situ

**Figure 5:**
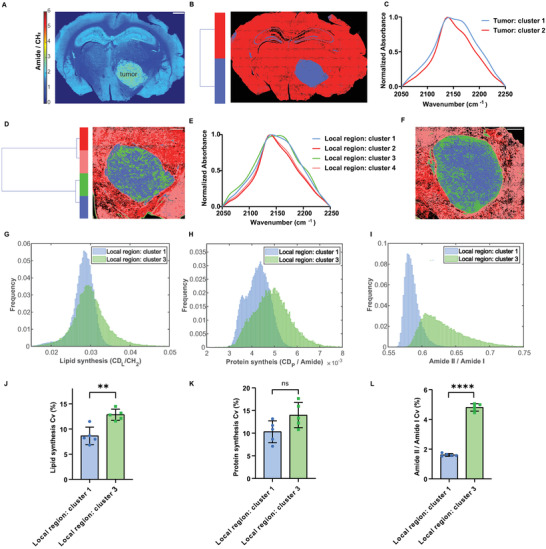
Revealing intratumor metabolic heterogeneity of glioblastoma in situ. A) Amide over CH_2_ ratio plot of the same tissue section, highlighting the margin of the tumor. Scale bar: 1 mm. B) HCA clustering of glioblastoma‐embedded mouse brain section. C) Spectral centroids from Figure 5B. D) HCA clustering of the local tumor region in Figure 5A, reflecting intratumor metabolic heterogeneity. Scale bar: 500 µm. E) Spectral centroids from Figure 5D. F) HCA clustering results of another GBM‐contained brain tissue section. Scale bar: 500 µm. G) Histogram of lipid synthesis of the segmented clusters from Figure 5D, described by the ratio of CD_L_ over CH_2_. H) Histogram of protein synthesis of segmented clusters from Figure 5D, described by the ratio of CD_P_ over amide. I) Histogram of the ratio of Amide II over Amide I of segmented clusters in Figure 5D. J) *C*_v_ values of lipid synthesis of segmented cluster 1 and cluster 3 (mean ± std, *n* = 5 groups). ** represents a P value <0.01 (P value = 0.0019). (K) Cv values of protein synthesis of segmented cluster 1 and cluster 3 (mean ± std, *n* = 5 groups). The symbol ns represents no significant difference (P value = 0.0555); L) *C*_v_ values of amide ratio of segmented cluster 1 and cluster 3 (mean ± std, *n* = 5 groups). **** indicates a *P* value < 0.0001. For the blue cluster, each group has 10 558 pixels; For the green cluster, each group has 5931 pixels.

We next applied our platform to capture metabolic aberration during diseases. Glioblastoma multiforme (GBM) are highly proliferative and heterogeneous tumors with altered metabolism.^[^ ^31^ ^]^ To study their metabolism, we collected the FTIR imaging data of GBM‐containing brain tissue samples, which were sliced from mice fed with 25% D_2_O for 15 days since U87 human glioma cells injection. Applying the same analysis strategy, it was found that with only 2 clusters in the HCA dendrogram, our method can already differentiate the tumor from the majority of brain tissue regions (**Figure** **5**B), which highlights the metabolic distinction of GBM compared with brain tissues. The metabolic profile mainly attributed to the GBM has a high shoulder peak proportion (~2180 cm^−1^, Figure 5C), which is consistent with the active protein synthesis of GBM.^[^ ^14^, ^32^ ^]^

Remarkably, when zooming into the GBM tumor region, we identified a unique cluster (green) that is largely located at the periphery of GBM tumor with a very high newly synthesized protein component in its metabolic profile (Figure 5E), which might be associated with the highly activated expression of Akt and mTOR in the GBM periphery region^[^ ^31^ ^]^ with an important role in stimulating protein synthesis and cell growth.^[^ ^33^ ^]^ Similar intratumoral metabolic heterogeneity was found in another GBM‐containing brain specimen (Figure 5F) with spectral information listed in Figure S3. To further characterize the metabolic activities of this unique green cluster at the periphery, we first extracted the average spectrum of these segmented clusters with intensity information in the CD region (Figure S3A, Supporting Information). Noteworthy, the average spectrum from the green cluster has very high intensities of the entire metabolic profile compared with other regions, suggesting upregulated metabolic activities in this region. A three‐concentric layers model for GBM has been put forward in the literature, classifying the GBM mass as core (necrotic), intermediate layer, and peripheral/hyper‐vascularized tumor area.^[^ ^34^ ^]^ The periphery of the mass is supposed to contain the highest proportion of differentiated GBM tumor cells with scant glioma stem cells, whereas the inner core is enriched in immature cells.^[^ ^35^ ^]^ Given that our green cluster is found to be largely located at the periphery of the GBM tumor and the measured metabolic activities are upregulated there, we speculate that our method supports this three‐layer model of the GBM mass and has revealed unique metabolic signatures of the peripheral layer.

Further insights can be obtained about this intratumoral metabolic heterogeneity. We plotted the histograms of lipid synthesis and protein synthesis for segmented green and blue clusters, which both locate inside the tumor. For lipid synthesis, it is found that the green cluster likely representing the peripheral layer has a higher mean value and an evidently larger variance (Figure 5G). This result is further confirmed by the *C*_v_ value characterization, which is considered to be a normalized measure of dispersion (Figure 5J). For protein synthesis, the green cluster also showed a larger mean value and a larger Cv value (Figure 5H,K). These results first proved the active metabolic activities in this peripheral region including both lipid and protein synthesis compared with the other tumor regions. Moreover, the large Cv values suggested a large heterogeneity for the lipid and protein synthesis localized in the green cluster region, which might be due to a mixture of different cell types there. Besides metabolic information, we also plotted the histogram of the ratio of amide II over amide I for these two clusters (Figure 5I). The green cluster has a larger mean and very broad distribution of the amide ratio values. Since the intensity of this amide ratio is associated with the changes of protein secondary structures,^[^ ^36^ ^]^ our result indicates the more diversified protein secondary structures localized within the green cluster as well.

## 3 Discussion

In conclusion, we developed a platform integrating FTIR imaging, D_2_O labeling, and an effective data analysis workflow for mapping all‐around metabolic atlas. Leveraging multivariate analysis, we identified the characteristic metabolic profile of individual organ and thus created a metabolic tissue atlas map, which highlighted the unique metabolic activities of each organ and revealed their possible metabolic connections. By harnessing the HCA clustering method, we spatially resolved intra‐tissue metabolic signatures of the mice brain, including the classic landmarks of the cerebellum, forebrain, and olfactory bulb. Applying this analysis strategy to development and diseases, we captured the metabolic profile changes during brain development and revealed the intratumoral metabolic heterogeneity of GBM, which might bring new insights into the highly heterogeneous nature of GBM.

Our success in applications of advanced data analysis strategies stems from the high‐quality C—D spectral data from FTIR, which is advantageous in providing high‐quality spectra with desired spectral resolutions in a time‐efficient way. Another reason for the successful implementations of t‐SNE and UMAP is the similar data structure between vibrational spectrum and gene sequencing. Indeed, the normalized intensity at each IR frequency can just be treated as the normalized expression level of a particular gene in the data analysis. Thus, it is foreseen that various popular algorithms developed for gene sequencing analysis^[^ ^37^ ^]^ can be directly transferred to our platform.

Several future lines of developments can be anticipated. The generalizability of workflow makes it readily compatible with other infrared imaging modalities. For example, subcellular metabolic signatures can be explored through the coupling of our method with MIR photothermal imaging,^[^ ^38^ ^]^ MIR photoacoustic imaging,^[^ ^39^ ^]^ infrared‐optical hybrid microscopy,^[^ ^40^ ^]^ or AFM‐IR.^[^ ^41^ ^]^ In vivo metabolic imaging is promising using MIR photoacoustic^[^ ^39^, ^42^ ^]^ and far‐field resolved IR spectroscopy,^[^ ^43^ ^]^ which are non‐invasive and free of water background. Regarding metabolic probes, although D_2_O is a universal and economical metabolic probe for in vivo labeling, other reported IR‐active metabolic probes including azido‐palmitic acid, ^13^C amino acids,^[^ ^11^ ^]^ and deuterated arachidonic acid^[^ ^44^ ^]^ can also be applied to animals to study more specific activities of designated metabolites, contributing to the spatial metabolomics derived from MIR metabolic imaging. Furthermore, drug design can be benefited from our method, especially for cancer therapy. By administrating drugs to metabolic probe‐labeled animals, intratumoral metabolism alterations after drug treatment can be analyzed, which will provide direct and visualizable evaluations of drug effectiveness.

## 4 Experimental Section

### Materials

Deuterium oxide (151 882) and methanol (34 860) were purchased from Sigma‐Aldrich. Proteinase K (FEREO0491) was purchased from Fisher‐Scientific. CaF_2_ substrates (CAFP25‐1, CAFP13‐1, and CAFP‐76‐26‐1U) were purchased from Crystran. No unexpected or unusually high safety hazards were encountered.

### Mouse D_2_O Labeling and GBM Tumor Xenograft

The animal protocol for mice studies was approved by Columbia University Institutional Animal Care and Use Committee (IACUC) (AC‐AABD1552). All experiments using mice were conducted following the ethical regulations of Columbia University IACUC. Wild‐type ≈3–4‐month female adult C57BL/6J mice were purchased from the Jackson Laboratory. For D_2_O labeling, 25% D_2_O in drinking water was given to the mice for 35 days for either wild‐type 3–4‐month‐old adult mice or pup mice (P35) delivered from breeding.

To establish the glioblastoma xenograft model, U87MG human glioma cells were implanted intracranially on nude mice (J:NU, Jackson Laboratory). The mice were anesthetized and stabilized in a stereotaxic instrument (David Kopf Instruments), and then the head skin was cut open to expose the skull bone. A small section (2 mm in diameter) on the skull frontal region was ground with a dental drill (Braintree Scientific) until it became transparent and soft. 1.5 × 10^5^ U87MG tumor cells in 3 µL were injected into the frontal region of the cerebral cortex in 5 min using a 1.5‐mm glass via the small section. Mouse head skin was then recovered with silk sutures (Harvard Apparatus) after implantation. Two mice were sacrificed at 15 d post xenograft. During tumor progression, the animals were monitored daily for any sign of morbidity, including weight loss, appetite loss, dehydration, inability to maintain balance, or unresponsiveness to noxious external stimuli, such as tow‐pinch withdrawal test. For glioblastoma‐bearing mice, 25% D_2_O were provided for 15 days since tumor implantation. They were then sacrificed for imaging experiments.

For tissue slicing, the organs were first embedded in 6% agarose gel and then cut into 20 µm thin slices with a vibratome (Leica) in HBSS buffer. The collected tissue slices were washed twice with HBSS buffer and twice with dd‐H2O. The samples were then mounted on CaF_2_ substrates for overnight air drying.

### FTIR Imaging

Cary 620 Imaging FTIR equipped with an Agilent 670‐IR spectrometer and 128 × 128‐pixels FPA mercury cadmium telluride (MCT) detector was used in the transmission mode. Background spectra were collected on CaF_2_ substrates using 128–256 scans at 8 cm^−1^ spectral resolution. Sample spectra were recorded using 16–128 scans for tissues at 8 cm^−1^ spectral resolution. A 15 × IR objective (pixel size of 5.5 µm, 0.62 NA) and a 25 × IR objective (pixel size of 3.3 µm, 0.81 numerical aperture (NA)) were used for the imaging.

### Data Preprocessing

Data preprocessing was performed using both the commercial software Cytospec and home‐built MATLAB scripts with the following steps: 1) PCA noise reduction to denoise the spectra; 2) quality test to remove pixels with low SNR in both the fingerprint region and the C—D region; 3) rubber‐band baseline correction or RMieSC baseline correction for spectral correction; 4) min‐max data normalization for the whole spectrum; 5) spectral cut to extract the C—D region ranging from 2050 to 2250 cm^−1^ from the whole dataset; 6) min‐max data normalization of the C—D region (C—D normalization, optional depending on the next‐step data analysis).

### 2D Scatter Graph Plot

To construct the 2D scatter graph, 5000 ratio values of CD_L_/CH_2_ and CD_P_/amide were randomly selected for each organ. These paired ratio values of each organ were then plotted on the 2D graph using MATLAB with solid lines indicating error ellipse with 99% confidence. Color coding was used to separate the data points from different organs. Overall, 40 000 data points (5000 × 8) were plotted simultaneously on the same 2D graph representing 8 organs.

### t‐SNE Analysis and Inter‐Organ Spearman Network Construction

For the t‐SNE analysis, 5000 spectra of the whole C—D spectra of each organ were randomly selected and constitute an integrated dataset of 40 000 spectra for 8 tissues. This integrated dataset was used as the input of t‐SNE. The C—D spectra were normalized in the C—D region to reveal macromolecule‐specific metabolic details in the metabolic profiles. Pairwise Spearman correlation coefficients of these 5000 C—D spectra for eight organs were calculated referencing the reported methods in single‐cell transcriptomics^[^ ^38^ ^]^ where each wavenumber intensity value was treated as a gene expression value. Spearman correlation was chosen over Pearson correlation because the correlations between C—D spectra of different tissues are not necessarily linear. Edges with coefficient values greater than 0.95 were considered significant and were kept in the network, as the correlations of different C—D spectra were quite strong. All these analysis methods were performed using home‐built MATLAB scripts.

### HCA Clustering and Intra‐Tissue Spearman Network Construction

HCA clustering was performed on normalized C‐D spectra using Euclidean distance and Ward's linkage method. For the intra‐tissue Spearman network, 5000 normalized C—D spectra of each segmented cluster from 3 brain tissues were randomly selected and used to calculate the Spearman correlation coefficients of C—D spectra between clusters. Edges with coefficient values greater than 0.95 were considered significant and were kept in the network. All these analysis methods were performed using home‐built MATLAB scripts.

### UMAP Analysis, SAM Score Calculation, and Intra‐Tissue Histogram Plots

Similar to the aforementioned approach, 5000 normalized C—D spectra were randomly chosen from each segmented cluster from pup and adult cerebellum tissues or pup and adult brain tissues and then used as the input of UMAP. For SAM score calculation, the metabolic profiles of pup mice were used as the test spectra and adult mice (Figure 3B) were used as the reference spectra, respectively. The calculated SAM score values from left to right in Figure 4D are 0.05475, 0.2101, 0.1906, 0.1589, 0.06924, 0.07017 0.08630, 0.1409, 0.05122 accordingly. The calculation was performed using the SAM function in the MATLAB hyperspectral imaging toolbox. As for the protein synthesis, lipid synthesis, and amide ratio histograms, the HCA clustering results were used as the masks to extract the ratio values of CD_L_/CH_L_, CD_P_/Amide, and amide II/amide I of all the pixels in each segmented cluster. Outliers of the ratio values were removed for the final histogram plots. For CD_L_/CH_L_, the mean ± std for the histograms of blue and green clusters are 0.0281 ± 0.0028 and 0.0299 ± 0.0043. For CD_P_/Amide, the mean ± std for the histograms of blue and green clusters are 0.0042 ± 0.0005 and 0.0049 ± 0.0008. For amide II/amide I, the mean ± std for the histograms of blue and green clusters are 0.5848 ± 0.0092 and 0.6303 ± 0.0291.

### Statistical Analysis

For the one‐way ANOVA tests of the metabolic ratio values of different organs and tissues (Table S1, Supporting Information), preprocessing was performed by removing the outliers of ratio values of either the CD_L_/CH_2_ group or the CD_P_/amide group in MATLAB. Then 5000 ratio values of either CD_L_/CH_2_ or CD_P_/amide were randomly selected. One‐way ANOVA tests were performed using the Prism software. The specific P values were listed in Table S1, Supporting Information.

For the Cv values of intratumoral metabolic heterogeneity analysis, the pixels from the segmented clusters were randomly split into 5 groups. Cv values were thus calculated based on these 5 groups for each cluster using MATLAB and presented in the mean ± std format. For the blue cluster, each group has 10 558 pixels; for the green cluster, each group has 5931 pixels. In Figure 5J of lipid synthesis, the specific mean values ± std values of Cv values for the blue and green clusters are 8.653 ± 1.741 and 12.83 ± 1.111 respectively; in Figure 5K of protein synthesis, the specific mean values ± std values for the blue and green clusters are 10.30 ± 2.412 and 13.98 ± 2.776 respectively; in Figure 5L of amide ratio, the specific mean values ± std values for the blue and green clusters are 1.611 ± 0.0943 and 4.802 ± 0.2482 respectively. One‐way ANOVA test was performed on the C_V_ values using the Prism software with P values listed in Figure 5J–L.

## Conflict of Interest

The authors declare no conflict of interest.

## Supporting information

Supporting Information

## Data Availability

The data that support the findings of this study are available from the corresponding author upon reasonable request.
