# Supplementary material for: Towards Mapping Mouse Metabolic Tissue Atlas by Mid‐Infrared Imaging with Heavy Water Labeling
Source: Adv Sci (Weinh). 2022 Mar 23;9(15):2105437. doi: 10.1002/advs.202105437 (PMC9131428; doi:10.1002/advs.202105437)
Supplement: Supplementary file 1 — Supporting Information [file ADVS-9-2105437-s001.pdf]

Supporting Information

Title: Towards mapping mouse metabolic tissue atlas by mid-infrared imaging with heavy water labeling

*Xinwen Liu, Lixue Shi, Lingyan Shi, Mian Wei, Zhilun Zhao and Wei Min**

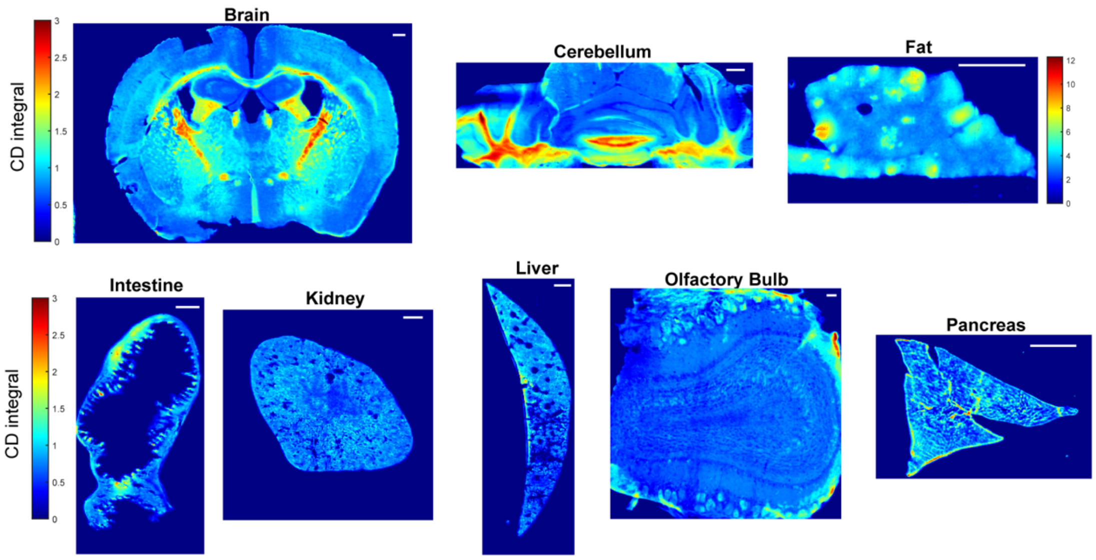

Figure S1. C-D integral images (the integral over the entire C-D region) of eight tissues used in quantitative metabolic activities analysis. The color bar of all the tissues except adipose tissue (fat) was ranged from 0-3. For fat, because of its rather large C-D signal, the color bar range was chosen from 0-12. Scale bar: for all the tissues except the olfactory bulb, the scale bar is 500 μm; for olfactory bulb, the scale bar is 100 μm.

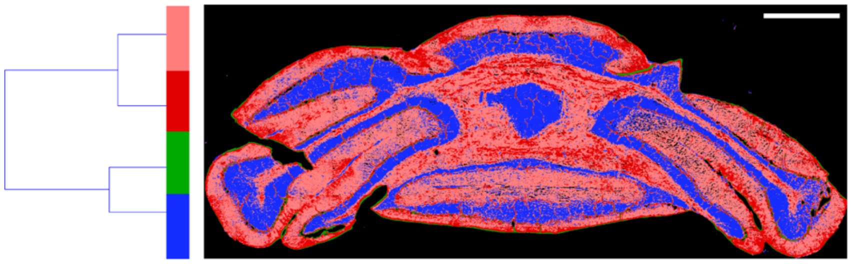

Figure S2. HCA clustering results of an adult cerebellum tissue section without C-D normalization. Scale bar: 1mm.

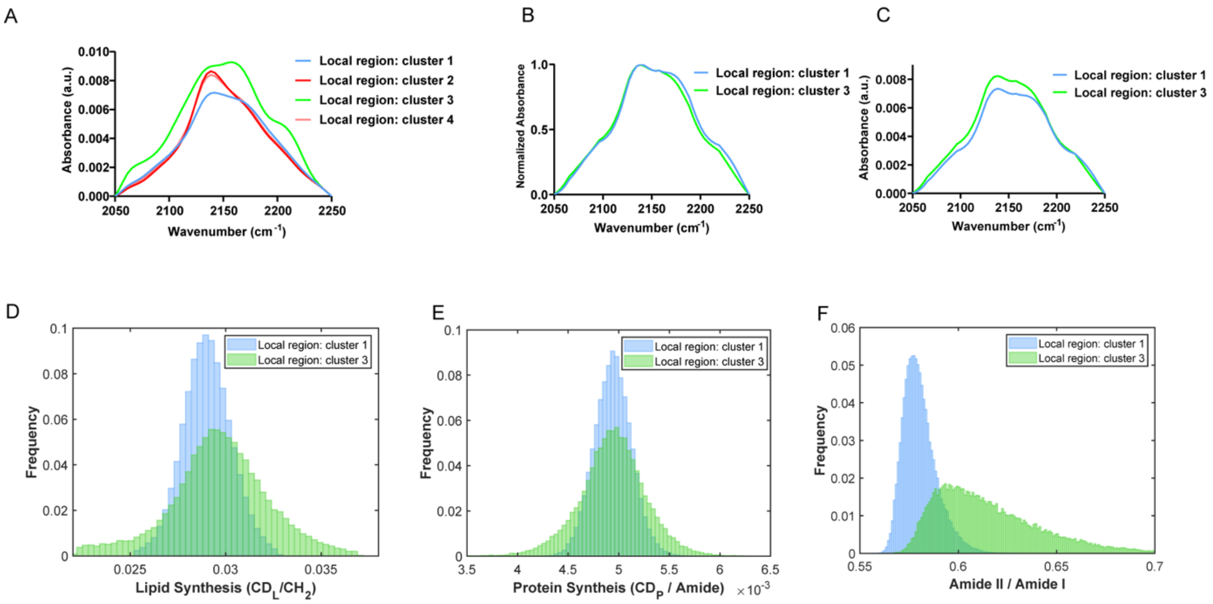

Figure S3. Metabolic heterogeneity characterization of GBM-contained brain tissue sections. (A) Average intensity spectra of segmented clusters from figure 5D. (B) Spectral centroids from figure 5F. (C) Average intensity spectra of segmented clusters from figure 5F. (D) Histogram of lipid synthesis of the segmented clusters from figure 5F, described by the ratio of CD_L over CH_2. The mean ± std for the blue and green histograms are 0.0290 ± 0.0013 and 0.0291 ± 0.0036. (E) Histogram of protein synthesis of segmented clusters from figure 5F, described by the ratio of CD_P over amide. The mean ± std for the blue and green histograms are 0.0049 ± 0.000019 and 0.0050 ± 0.000033. (F) Histogram of the ratio of Amide II over Amide I of segmented clusters in figure 5F. The mean ± std for the blue and green histograms are 0.5800 ± 0.0078 and 0.6138 ± 0.0244.

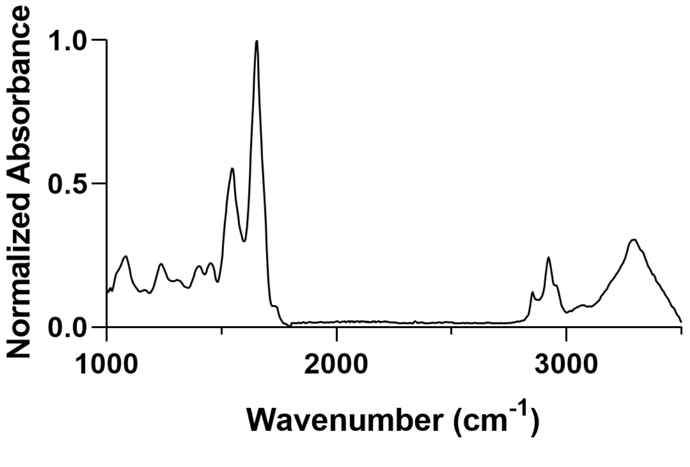

Figure S4. Control IR spectrum of tissue specimens without D_2O labeling

Table S1 ANOVA test results of ratio value groups from different organs and tissues

CD_L / CH_2

	Summary	P value
Br vs. CB	ns	>0.9999
Br vs. Fat	**	0.0027
Br vs. Intestine	****	<0.0001
Br vs. Kidney	**	0.0031
Br vs. Liver	****	<0.0001
Br vs. OB	ns	>0.9999
Br vs. Pancreas	*	0.0112
CB vs. Fat	**	0.0055
CB vs. Intestine	****	<0.0001
CB vs. Kidney	**	0.0062
CB vs. Liver	****	<0.0001
CB vs. OB	ns	0.9996
CB vs. Pancreas	*	0.0219
Fat vs. Intestine	****	<0.0001
Fat vs. Kidney	ns	>0.9999
Fat vs. Liver	****	<0.0001
Fat vs. OB	**	0.0014
Fat vs. Pancreas	ns	0.9994
Intestine vs. Kidney	****	<0.0001
Intestine vs. Liver	ns	0.8085
Intestine vs. OB	****	<0.0001

CD_P / amide

	Summary	P value
Br vs. CB	ns	0.8657
Br vs. Fat	ns	0.4575
Br vs. Intestine	****	<0.0001
Br vs. Kidney	*	0.0208
Br vs. Liver	****	<0.0001
Br vs. OB	ns	0.9927
Br vs. Pancreas	ns	0.4430
CB vs. Fat	ns	0.9964
CB vs. Intestine	****	<0.0001
CB vs. Kidney	***	0.0005
CB vs. Liver	****	<0.0001
CB vs. OB	ns	0.4060
CB vs. Pancreas	*	0.0300
Fat vs. Intestine	****	<0.0001
Fat vs. Kidney	****	<0.0001
Fat vs. Liver	****	<0.0001
Fat vs. OB	ns	0.1178
Fat vs. Pancreas	**	0.0050
Intestine vs. Kidney	****	<0.0001
Intestine vs. Liver	**	0.0056
Intestine vs. OB	****	<0.0001

Intestine vs. Pancreas	****	<0.0001
Kidney vs. Liver	****	<0.0001
Kidney vs. OB	**	0.0016
Kidney vs. Pancreas	ns	0.9997
Liver vs. OB	****	<0.0001
Liver vs. Pancreas	****	<0.0001
OB vs. Pancreas	**	0.0061

Intestine vs. Pancreas	****	<0.0001
Kidney vs. Liver	****	<0.0001
Kidney vs. OB	ns	0.1261
Kidney vs. Pancreas	ns	0.7944
Liver vs. OB	****	<0.0001
Liver vs. Pancreas	****	<0.0001
OB vs. Pancreas	ns	0.8910

(Br represents the forebrain, CB represents the cerebellum, OB represents the olfactory bulb)
